# *Serratia marcescens* peritonitis in a continuous ambulatory peritoneal dialysis patient: A case report and literature review

**DOI:** 10.1016/j.idcr.2026.e02764

**Published:** 2026-09-19

**Authors:** Orhan Altınyüzük, Melike Hazal Umut, Bartu Ediz, Meral Meşe, Serkan Feyyaz Yalın, Ergün Parmaksız

**Affiliations:** a Department of Internal Medicine, Kartal Dr. Lütfi Kırdar City Hospital, University of Health Sciences, İstanbul, Turkey; b Department of Nephrology, Kartal Dr. Lütfi Kırdar City Hospital, University of Health Sciences, İstanbul, Turkey

**Keywords:** *Serratia marcescens*, Peritonitis, Continuous ambulatory peritoneal dialysis (CAPD), Gram-negative peritonitis, Catheter removal, Case report

## Abstract

**Introduction:**

Peritonitis caused by *Serratia marcescens* in patients undergoing continuous ambulatory peritoneal dialysis is a rare but clinically significant condition. As an opportunistic gram-negative pathogen with notable genomic plasticity and biofilm-forming capacity, *Serratia marcescens* frequently leads to catheter loss and treatment failure. This case highlights the diagnostic challenges, antibiotic management strategy, and the clinical course leading to catheter removal in a high-risk continuous ambulatory peritoneal dialysis patient.

**Case presentation:**

A 61-year-old woman with stage 5 chronic kidney disease on continuous ambulatory peritoneal dialysis for four years presented with abdominal pain, nausea, fever (38.3°C), and cloudy dialysate. Dialysate white blood cell count was markedly elevated at 5600/mm³ with neutrophil predominance. Peritonitis was diagnosed per International Society for Peritoneal Dialysis criteria. Peritoneal fluid culture grew *Serratia marcescens* susceptible to meropenem, piperacillin/tazobactam, ciprofloxacin, ceftazidime, imipenem, and trimethoprim-sulfamethoxazole, but resistant to amikacin, amoxicillin-clavulanate, and ampicillin. Empirical therapy with piperacillin/tazobactam and teicoplanin was replaced by intravenous plus intraperitoneal meropenem combined with teicoplanin. Despite a partial decrease in dialysate cell count, persistent fever and inadequate acute phase reactant response led to catheter removal after approximately 21 days of targeted therapy. Following catheter removal and 14 additional days of antibiotic treatment (35 days total), the patient recovered fully and was transitioned to hemodialysis.

**Conclusion:**

This case underscores the aggressive nature of *Serratia marcescens* peritonitis in continuous ambulatory peritoneal dialysis patients and highlights the importance of prompt empirical antibiotic therapy, early culture-directed treatment escalation, and timely catheter removal decisions. Intraperitoneal plus intravenous meropenem represents a rational therapeutic strategy, yet catheter salvage may not always be achievable. Clinicians must maintain a high index of suspicion for catheter removal in refractory gram-negative peritonitis.

## Introduction

Approximately 800 million people worldwide suffer from chronic kidney disease (CKD), with around 3.5 million receiving hemodialysis and approximately 200,000 undergoing peritoneal dialysis (PD) [1]. Peritonitis remains one of the most serious complications of PD, accounting for significant morbidity, technique failure, and mortality. Among gram-negative pathogens, which are responsible for approximately 20–30% of PD-associated peritonitis cases [1], *Serratia marcescens* is a particularly uncommon but clinically challenging organism.

As an opportunistic, nosocomial gram-negative bacillus, *S. marcescens* is capable of transmission via urinary, gastrointestinal, respiratory, and direct-contact routes [2]. Within the peritoneal cavity, its virulence is substantially enhanced by notable genomic plasticity, intrinsic beta-lactamase production, and an exceptional capacity to form resilient biofilms on catheter surfaces [3]. These pathophysiological properties collectively impede antibiotic penetration, promote treatment failure, and frequently result in permanent catheter loss [[3], [4], [5], [6]]. To date, 23 genomic variants of *S. marcescens* have been described, highlighting its evolutionary capacity to adapt to antimicrobial stress [3].

The International Society for Peritoneal Dialysis (ISPD) 2022 guidelines emphasize that gram-negative peritonitis carries a significantly worse prognosis than gram-positive peritonitis, demonstrating substantially higher rates of mandatory catheter removal and mortality [1]. Clinical outcomes are heavily time-dependent; for every one-hour delay in initiating appropriate antibiotic therapy following hospital presentation, the risk of permanent PD technique failure or patient death increases by 5.5% [1].

Because published literature on *S. marcescens* peritonitis in PD patients remains scarce, clinical consensus regarding optimal management is limited. While a minority of documented cases achieve an antibiotic-only cure, the historical majority culminate in adverse outcomes, including permanent catheter loss or mortality [[4], [5], [6], [7], [8], [9], [10], [11]]. This case report aims to address this gap in the literature by detailing the clinical course, microbiological profile, and therapeutic challenges of a *S. marcescens* peritonitis episode in a continuous ambulatory peritoneal dialysis patient. To ensure the highest standard of verification, clarity, and educational value, this report has been structured in strict accordance with the CARE (CAse REports) guidelines [12], and explicit written informed consent was obtained from the patient prior to publication.

## Case presentation

A de-identified 61-year-old woman presented to our outpatient and inpatient nephrology service. She had been managed with CAPD for approximately four years for stage 5 CKD (end-stage renal disease). No family history of immune deficiency or hereditary renal disease was documented. Psychosocial history was unremarkable; the patient lived at home and performed CAPD exchanges independently.

Her primary presenting concerns were diffuse abdominal pain and nausea evolving over approximately one to two days. On direct questioning, she reported similar prior episodes (2–3 per year) managed with antibiotics during inpatient stays; these had been treated empirically with piperacillin/tazobactam and tigecycline, and specific pathogens from previous episodes were not available for comparison. No history of recent surgical procedures, gastrointestinal illness, urinary tract infection, or healthcare-associated procedures beyond routine CAPD was identified. There was no documented immunosuppressive therapy.

On physical examination, body temperature was 38.3°C. Blood pressure and heart rate were within normal limits. Abdominal examination revealed tenderness localized to the periumbilical and catheter-adjacent region. No guarding, rebound tenderness, or signs of peritoneal irritation were present. Bowel sounds were present. The CAPD catheter exit site was intact, and clinical palpation of the subcutaneous tunnel revealed no erythema, induration, tenderness, or purulent discharge, providing no initial physical evidence of periluminal or tunnel infection.

An electrocardiogram was performed to exclude acute coronary syndrome as a cause of abdominal pain; no diffuse ischemic changes were identified.

The dialysate fluid drained from the peritoneal catheter was visibly cloudy. Laboratory analysis of the dialysate demonstrated a white blood cell count of 5600/mm³ with neutrophil predominance (>50% polymorphonuclear cells), consistent with infectious peritonitis per ISPD diagnostic criteria [1]. Peripheral blood WBC was 18,000/mm³ and CRP was 350 mg/L; erythrocyte sedimentation rate was also elevated. Table 1 summarizes the chronological sequence of clinical events, diagnostic findings, and therapeutic decisions throughout this episode of care.

**Table 1:** Clinical timeline of the current episode.

**Day**	**Event**
Day 0	Admission: abdominal pain, nausea, fever 38.3°C; cloudy dialysate observed; dialysate WBC 5600/mm³; peripheral WBC 18,000/mm³, CRP 350 mg/L; empirical piperacillin/tazobactam 3 × 2.25 g IV + teicoplanin IV started; infectious diseases consulted
Day 2–3	Peritoneal fluid culture result: *Serratia marcescens* isolated (no mixed growth)
Day 3–4	Antibiogram results: susceptible to meropenem, pip/tazo, imipenem, ciprofloxacin, ceftazidime, TMP-SMX; resistant to amikacin, amoxicillin-clavulanate, ampicillin, cefoxitin. Regimen changed to meropenem 0.5 g IV + 0.5 g IP once daily; teicoplanin continued
Day 21	Despite treatment, inadequate acute-phase reactant response (CRP 300 mg/L, WBC 12,000/mm³, procalcitonin 1 ng/mL); dialysate WBC trended from 5600 → 50/mm³ (fluctuating course); persistent fever; repeat peritoneal fluid culture negative for *S. marcescens* despite ongoing systemic inflammation
Day 21	Decision to remove peritoneal catheter (culture-negative effluent but persistently elevated systemic markers); permanent hemodialysis catheter inserted; hemodialysis initiated (3 sessions/week)
Day 21–35	14 additional days of antibiotic therapy post-catheter removal (total 35-day course)
Day 35	Fever resolved; APR normalized; abdominal pain and nausea resolved; dialysate WBC 50/mm³; patient discharged

The diagnosis of infectious peritonitis was established as all three-baseline clinical, cytological, and microbiological criteria of the ISPD 2022 guidelines were fully met [1]. Peritoneal effluent was sent for cell count and differential, Gram stain, aerobic and anaerobic cultures, and antibiotic susceptibility testing. Blood cultures were obtained to exclude bacteremia. Abdominal ultrasound and plain radiography were performed to rule out mechanical complications.

Upon admission, peritonitis was clinically suspected based on cloudy dialysate, abdominal pain, fever, and markedly elevated dialysate cell count. Infectious diseases were consulted immediately. Empirical therapy was initiated with piperacillin/tazobactam 2.25 g IV three times daily and teicoplanin IV to provide combined gram-negative and gram-positive coverage, consistent with current ISPD recommendations for empirical broad-spectrum therapy [1].

Following culture and susceptibility results confirming *S. marcescens* peritonitis, piperacillin/tazobactam was discontinued. Peritoneal fluid culture yielded *S. marcescens* as a pure isolate (no mixed growth). The isolate was susceptible to carbapenems (ertapenem ≤0.12 μg/mL, meropenem ≤0.25 μg/mL, imipenem 0.5 μg/mL), third-generation cephalosporins (ceftriaxone ≤0.25 μg/mL, ceftazidime ≤0.5 μg/mL), ciprofloxacin (0.12 μg/mL), piperacillin/tazobactam (≤4.0 μg/mL), and trimethoprim/sulfamethoxazole (≤20.0 μg/mL). Intrinsic or acquired resistance was noted for amikacin (4.0 μg/mL), amoxicillin/clavulanic acid (8.0 μg/mL), ampicillin (16.0 μg/mL), and cefoxitin (8.0 μg/mL). The antimicrobial susceptibility profile is detailed in Table 2. Meropenem was selected as the definitive agent based on the antibiogram, the organism's susceptibility profile, and its well-established pharmacodynamic properties: carbapenems exhibit high affinity for penicillin-binding proteins (PBPs) and resist hydrolysis by many beta-lactamase enzymes [3,10,11]. The regimen was: meropenem 0.5 g IV once daily plus meropenem 0.5 g IP once daily, combined with continuation of teicoplanin IV. The dual IV and IP administration route was chosen to maximize both systemic and intraperitoneal drug concentrations at the infection site [11].

**Table 2:** Antibiogram of *Serratia marcescens* isolated from peritoneal fluid culture.

**Antimicrobial Agent**	**MIC Value (µg/mL)**	**Clinical Interpretation (S / R)**
**Susceptible (S)**		
Meropenem	≤ 0.25	Susceptible (S)
Imipenem	0.5	Susceptible (S)
Ceftazidime	≤ 0.5	Susceptible (S)
Ciprofloxacin	0.12	Susceptible (S)
Piperacillin/Tazobactam	≤ 4.0	Susceptible (S)
Trimethoprim/Sulfamethoxazole (TMP-SMX)	≤ 20.0	Susceptible (S)
Ertapenem	≤ 0.12	Susceptible (S)
Ceftriaxone	≤ 0.25	Susceptible (S)
**Resistant (R)**		
Amikacin	4	Resistant (R)
Amoxicillin/Clavulanic Acid	8	Resistant (R)
Ampicillin	16	Resistant (R)
Piperacillin/Clavulanic Acid	8	Resistant (R)
Cefoxitin	8	Resistant (R) — phenotypic marker of AmpC inducibility

After approximately 21 days of targeted meropenem-based therapy, the patient continued to exhibit persistent fever and inadequate normalization of acute-phase reactants (APR: CRP 300 mg/L, WBC 12,000/mm³, procalcitonin 1 ng/mL) despite a reduction in dialysate WBC from 5600 to 50/mm³ (with a fluctuating course). A repeat peritoneal fluid culture obtained at this point was negative for *S. marcescens*, indicating that systemic inflammation persisted despite apparent microbiological clearance of the effluent. Given this refractory picture, the peritoneal catheter was removed in accordance with ISPD guidelines recommending catheter removal for refractory gram-negative peritonitis [1]. Timing was influenced by patient-centered factors: the patient initially resisted catheter extraction given the anticipated transition to hemodialysis, prompting a continued trial of antimicrobial therapy while serial subcutaneous and exit-site ultrasonography assessed salvage feasibility; this showed no tunnel infection, pericatheter collection, or cuff involvement. Given the ongoing lack of improvement, the patient was counseled on the pathogen's refractory nature and consented to catheter removal. A permanent hemodialysis catheter was inserted, and the patient was enrolled in a thrice-weekly hemodialysis program. Antibiotic therapy was continued for 14 additional days following catheter removal, completing a total treatment course of 35 days.

Following catheter removal, the patient's clinical condition improved rapidly. Body temperature normalized within the first few days post-removal. Abdominal pain and nausea resolved completely. Dialysate cell count at the time of discharge was 50/mm³. Systemic inflammatory markers normalized, and APR response was achieved. The patient was successfully transitioned to hemodialysis with a well-functioning permanent catheter and was enrolled in a 3-session-per-week program. At discharge after completing the 35-day antibiotic course, no adverse events related to antimicrobial therapy (nephrotoxicity, hepatotoxicity, drug-related rash) were documented. Adherence to the prescribed antibiotic regimen during hospitalization was confirmed through nursing records.

## Discussion

This case report describes a rare and clinically challenging episode of *S. marcescens* peritonitis in a CAPD patient with recurrent peritonitis, ultimately resulting in catheter loss and modality transition to hemodialysis despite appropriate antibiotic therapy. Its clinical significance lies not only in the rarity of *S. marcescens* as a CAPD peritonitis pathogen, but also in the illustration of the organism's capacity to drive treatment failure even when susceptibility testing indicates an adequate antibiotic regimen.

Peritonitis remains the leading cause of technique failure and a major contributor to morbidity and mortality among CAPD patients [8]. While gram-positive organisms, particularly *Staphylococcus epidermidis*, predominate in PD-associated peritonitis, gram-negative organisms account for 20–30% of cases and are consistently associated with worse outcomes, including higher rates of catheter removal and mortality [1]. Among gram-negative pathogens, *S. marcescens* is one of the rarest isolated organisms, yet one of the most clinically threatening.

The pathogenicity of *S. marcescens* in the peritoneal cavity relates to two properties that were not directly demonstrated in our isolate but remain plausible contributors given the clinical course. First, the organism shows genomic plasticity, with 23 variants described to date and a recognized capacity for inducible AmpC beta-lactamase expression [3]; our isolate displayed no phenotypic ESBL pattern and was susceptible to all cephalosporins tested, though its resistance to cefoxitin is a recognized indirect marker of AmpC inducibility. Second, *S. marcescens* can form robust biofilms on catheter surfaces, undermining antibiotic penetration regardless of in vitro susceptibility [2,3]; biofilm could not be directly examined on the explanted catheter, as electron microscopy was unavailable at our institution. The clinical course is nonetheless consistent with a biofilm-related mechanism: a repeat peritoneal culture became negative for *S. marcescens* while fever and APR remained elevated, suggesting a catheter-associated reservoir not reflected in planktonic effluent counts. This discordance, rather than direct microbiological proof, is what guided our judgement that catheter removal was necessary despite technically adequate antimicrobial therapy [[4], [5], [6]].

In our patient, the initial empirical regimen of piperacillin/tazobactam and teicoplanin was appropriately broad-spectrum and aligned with ISPD 2022 guidelines [1]. However, the absence of adequate APR response prior to culture results was an early clinical indicator of potential failure. Once *S. marcescens* was identified, the isolate was in fact susceptible to ceftazidime and ciprofloxacin, narrower-spectrum options that antimicrobial stewardship principles would ordinarily favour. Meropenem was nonetheless selected given the severity and recurrent nature of the infection and the recognized potential for inducible AmpC-mediated resistance to emerge during cephalosporin therapy against *S. marcescens* and related Enterobacterales; current IDSA guidance on antimicrobial-resistant gram-negative infections similarly favours cefepime or a carbapenem over third-generation cephalosporins for serious infections with AmpC-inducible organisms, independent of baseline susceptibility [13]. This decision therefore rested on overall clinical status rather than antibiogram results alone, a trade-off against strict narrowest-spectrum stewardship that we judged justified by the patient's systemic inflammatory response and treatment-refractory history. Bhave et al. [11] similarly reported successful use of IP meropenem in a multidrug-resistant *S. marcescens* peritonitis case, suggesting that the IP route may be particularly valuable in maximizing intraperitoneal drug concentrations.

Comparison with previously published cases (Table 3) highlights important similarities and differences. Of the six previously reported cases in which catheter salvage was achieved without removal, treatment durations ranged from 14 to 29 days, and all involved organisms susceptible to the empirical or culture-directed regimens used [4,5,[7], [8], [9],^11^]. Notably, Xie et al. [5] reported a case where combination IP and IV therapy over 22 days achieved cure without catheter removal, while Yang et al. [8] required sequential escalation through four antimicrobial regimens over 29 days. The present case shares the escalation pathway but differs in that persistent systemic inflammation, rather than rising dialysate cell counts, was the primary indicator of treatment failure.

**Table 3:** Summary of published cases of peritoneal dialysis-associated *Serratia marcescens* peritonitis [5,8,9,11,[13], [14], [15]].

This clinical discordance between dialysate WBC normalization (from 5600 to 50/mm³) and persistent fever and elevated APR markers warrants comment. It likely reflects compartmentalized resolution of peritoneal inflammation while systemic infection or catheter-related biofilm reservoir persisted. This phenomenon underscores an important clinical teaching point: normalization of dialysate cell counts alone should not be interpreted as treatment success in *S. marcescens* peritonitis. Systemic markers and fever curves must be monitored in parallel [1].

The ISPD 2022 guidelines [1] recommend catheter removal for refractory peritonitis that fails to clinically improve within five days of appropriate antibiotic therapy. In our case, catheter removal was undertaken around day 21 of culture-directed therapy which is considered a timeline that may reflect appropriate clinical hesitancy given partial dialysate response, but also underscores the difficulty of the decision in real-world practice. Following catheter removal, rapid clinical improvement was observed, lending support to the view that the catheter itself served as a biofilm reservoir perpetuating systemic infection.

This case also reinforces the importance of timely empirical antibiotic initiation, given the well-documented time-dependence of outcomes in gram-negative PD peritonitis [1]. Cloudy dialysate, even without abdominal symptoms, almost invariably represents infectious peritonitis and must be treated presumptively [1].

The recurrent nature of peritonitis in our patient (2–3 episodes per year over four years) raises broader questions about patient-specific risk factors for *S. marcescens* acquisition. Environmental contamination, hygiene practices, healthcare-associated exposure, and immune compromise in the context of end-stage renal disease are all plausible contributing factors [2]. The exact route of infection in this case could not be established, which is a recognized limitation of retrospective case reporting.

Identifying the portal of entry is often challenging in *S. marcescens* peritonitis. The absence of exit-site or tunnel infection signs argues against periluminal tracking, and the lack of recent invasive gastrointestinal procedures argues against transluminal migration. The most likely route was therefore intraluminal, via touch contamination during dialysate exchanges, consistent with epidemiological data that most non-tunnel-related gram-negative peritonitis stems from breaches in aseptic technique.

Strengths of this report include a full antibiogram, comprehensive clinical description, and comparison with all available published cases. Limitations include its single-case design, the inability to determine the exact source of infection, the absence of molecular typing of the *S. marcescens* isolate, the inability to directly examine the explanted catheter for biofilm as electron microscopy was unavailable at our institution, and the lack of long-term hemodialysis follow-up data. Additionally, the delay in catheter removal (approximately 21 days of failing targeted therapy) may have prolonged the patient's course and could be considered suboptimal in retrospect.

## Conclusion

*Serratia marcescens* peritonitis in CAPD patients is a rare but severe complication characterized by biofilm formation, genomic plasticity, and a high propensity for catheter loss and technique failure. In this case, combined intravenous and intraperitoneal meropenem therapy reduced peritoneal cell counts but was insufficient to achieve systemic cure, ultimately necessitating catheter removal and hemodialysis transition after 35 days of antibiotic therapy. This case reinforces that in *S. marcescens* peritonitis, normal dialysate cell count does not guarantee clinical resolution; systemic inflammatory response must guide treatment decisions. Early and decisive catheter removal in the setting of refractory fever and elevated acute phase reactants may lead to better outcomes. Further prospective studies and multicenter registries are needed to establish standardized treatment protocols for this rare but life-threatening condition.

## CRediT authorship contribution statement

**Orhan Altınyüzük:** Writing – original draft, Resources, Project administration, Conceptualization. **Melike Hazal Umut:** Investigation. **Bartu Ediz:** Supervision, Investigation. **Meral Meşe:** Supervision, Investigation. **Serkan Feyyaz Yalın:** Supervision, Investigation, Funding acquisition. **Ergün Parmaksız:** Validation, Supervision, Investigation.

## Ethics declaration

Verbal informed consent to take part in the study and to publish the article was obtained from all participants or their legal representatives. "Written informed consent could not be obtained, as the patient could not be reached in person following discharge; the patient was subsequently contacted by telephone, and verbal informed consent for publication was obtained and documented in the medical record by the treating physician on May 15, 2026. Formal ethics committee approval was not required for this case report, as it did not involve any experimental intervention and all patient information was anonymized in accordance with institutional and international guidelines.". The privacy rights of participants have been observed.

This study was performed in compliance with relevant laws, regulatory frameworks and guidelines where the research took place. Ethics committee approval was not required under relevant laws and institutional guidelines. "This report describes the retrospective clinical course of a single patient and did not involve any experimental or investigational intervention beyond routine standard-of-care management. As such, it falls outside the scope of Turkey's Regulation on Clinical Trials (Klinik Araştırmalar Hakkında Yönetmelik; Official Gazette No. 28030, 19 August 2011, Ministry of Health), which governs prospective interventional clinical research and mandates ethics committee review specifically for such studies. This is consistent with the International Committee of Medical Journal Editors (ICMJE) recommendations and institutional practice at our center, under which single-patient case reports based on routine clinical care do not require formal ethics committee approval, provided patient confidentiality is maintained and informed consent for publication is obtained."

This research follows the CARE guidelines and the CARE checklist.

## Ethical approval statement

Ethical approval was not required for this case report, as no experimental interventions were performed, and all patient information was anonymized per institutional and international guidelines.

## Informed consent

Informed consent was obtained from the patient to participate in the current study.

## Statement of adherence to preprint policy

The authors declare that this paper has not been published as a preprint or in any other form elsewhere.

## Funding information

This research received no specific grants from any funding agency in the public, commercial, or not-for-profit sectors.

## Conflict of Interest Statement

The authors declare no conflicts of interest in preparing this paper.

## Declaration of Competing Interest

None.

## Data Availability

The datasets generated during and/or analyzed during the current study are available from the corresponding author upon reasonable request.
